# Elevated LysoGb3 Concentration in the Neuronopathic Forms of Mucopolysaccharidoses

**DOI:** 10.3390/diagnostics10030155

**Published:** 2020-03-13

**Authors:** Galina Baydakova, Alex Ilyushkina, Lidia Gaffke, Karolina Pierzynowska, Igor Bychkov, Agnieszka Ługowska, Grzegorz Wegrzyn, Anna Tylki-Szymanska, Ekaterina Zakharova

**Affiliations:** 1 Federal State Budgetary Institution, Research Centre for Medical Genetics, 115478 Moscow, Russia; baydakovag@gmail.com (G.B.); bychkov.nbo@gmail.com (I.B.); doctor.zakharova@gmail.com (E.Z.); 2 Department of Molecular Biology, University of Gdansk, 80-308 Gdansk, Poland; lidia.gaffke@phdstud.ug.edu.pl (L.G.); karolina.pierzynowska@ug.edu.pl (K.P.); grzegorz.wegrzyn@biol.ug.edu.pl (G.W.); 3 Department of Genetics, Institute of Psychiatry and Neurology, 02-957 Warsaw, Poland; alugipin@yahoo.com; 4 Department of Pediatric Nutrition and Metabolic Diseases, The Children’s Memorial Health Institute, 04-730 Warsaw, Poland; atylki@op.pl

**Keywords:** globotriaosylsphingosine, mucopolysaccharidoses, heparan sulfate, glycosaminoglycans, biochemical marker, inherited metabolic diseases

## Abstract

Mucopolysaccharidoses (MPSs) are a group of lysosomal storage disorders associated with impaired glycosaminoglycans (GAGs) catabolism. In MPS I, II, III, and VII, heparan sulfate (HS) cannot be degraded because of the lack of sufficient activity of the respective enzymes, and its accumulation in the brain causes neurological symptoms. Globotriaosylsphingosine (LysoGb3), the deacylated form of globotriaosylceramide (Gb3), is described as a highly sensitive biomarker for another lysosomal storage disease—Fabry disease. The connection between MPSs and LysoGb3 has not yet been established. This study included 36—MPS I, 15—MPS II, 25—MPS III, 26—MPS IV, and 14—MPS VI patients who were diagnosed by biochemical and molecular methods and a control group of 250 males and 250 females. The concentration of lysosphingolipids (LysoSLs) was measured in dried blood spots by high pressure liquid chromatography—tandem mass spectrometry. We have demonstrated that LysoGb3 concentration was significantly elevated (*p* < 0.0001) in untreated MPS I (3.07 + 1.55 ng/mL), MPS II (5.24 + 2.13 ng/mL), and MPS III (6.82 + 3.69 ng/mL) patients, compared to the control group (0.87 + 0.55 ng/mL). LysoGb3 level was normal in MPS VI and MPS IVA (1.26 + 0.39 and 0.99 + 0.38 ng/mL, respectively). Activity of α-galactosidase A (α-Gal A), an enzyme deficient in Fabry disease, was not, however, inhibited by heparan sulfate in vitro, indicating that an increase of LysoGb3 level in MPS I, MPS II, and MPS III is an indirect effect of stored MPSs rather than a direct result of impairment of degradation of this compound by HS. Our findings indicate some association of elevated LysoGb3 concentration with the neuronopathic forms of MPSs. The pathological mechanism of which is still to be studied.

## 1. Introduction

Mucopolysaccharidoses (MPSs) are a group of inherited metabolic disorders caused by a deficiency of lysosomal enzymes that take part in the degradation of glycosaminoglycans (GAGs): heparan sulfate (HS), dermatan sulfate (DS), keratan sulfate (KS), and hyaluronic acid. MPSs are inherited in an autosomal recessive trait except for MPSs type II, which is recessive X-linked. The total frequency of all types of MPSs is around 1.8–4.5 in 100,000 live births [1,2,3]. MPS I (Hurler syndrome), MPS II (Hunter syndrome), MPS III (Sanfilippo diseases), and MPS VII (Sly diseases) are diseases associated with a central nervous system (CNS) involvement. However, MPS I (Scheie) and MPS II mild forms can manifest without neurological symptoms. Neuronopathic MPSs are characterized by neurocognitive regression, developmental delay, behavioral and sleep disturbances, vision disturbances, and hearing-loss [4]. The brain changes include signs of brain atrophy, ventricular enlargement, abnormalities in the white matter and perivascular spaces, and compressive myelopathy [5,6]. The pathophysiology of neuronal damage in neuronopathic types of MPSs is related to the storage of undegraded GAGs, in particular, HS, and secondary toxic products such as GM2 and GM3 gangliosides, inflammatory cytokines, and reactive oxygen species [7].

Enzyme activity assay is the “gold standard” for diagnosing MPSs, but quantitative and qualitative analysis of urinary (or blood) GAGs is used as a primary screening test for most types of MPSs [8]. Levels of different GAGs increase depending on the type of MPSs; however, they can also be elevated in mucolipidoses (MLs). Sensitivity and specificity of measurement of multiple GAGs by high pressure liquid chromatography–tandem mass spectrometry (LC–MS/MS) are high, and this method could be used for the diagnosis of severe forms of MPSs and ML and, potentially, also for monitoring therapeutic effects. However, it should be noted that MPS patients with attenuated phenotypes may have false-negative results due to lower GAG levels [9]. The determination of GAGs in dried blood spot (DBS) is a rather complicated method, which is not carried out in all laboratories [10].

The availability of new methods of treatment for MPSs (hematopoietic stem cell transplantation (HSCT) and enzyme replacement therapy (ERT)), and ongoing studies of other novel therapeutic options (e.g., substrate reduction therapy and gene therapy), require the development of new effective approaches for their early diagnosis. ERT was approved for MPS I, MPS II, MPS IVA, MPS VI, and MPS VII, and it is not yet available for MPS IVB and MPSs III, but studies are ongoing, as does the search for sensitive and specific markers [11,12].

In this study, we determined LysoGb3 concentration in DBSs in MPS I, MPS II, MPS III, MPS IV, and MPS VI patients and showed that this metabolite could be a potential secondary biomarker for the neuronopathic MPSs forms.

## 2. Results

We measured LysoGb3 concentrations in the DBS samples derived from a total of 116 untreated patients with different types of MPSs, 51 MPS I patients treated with ERT, and 500 healthy controls.

**Figure 1:**
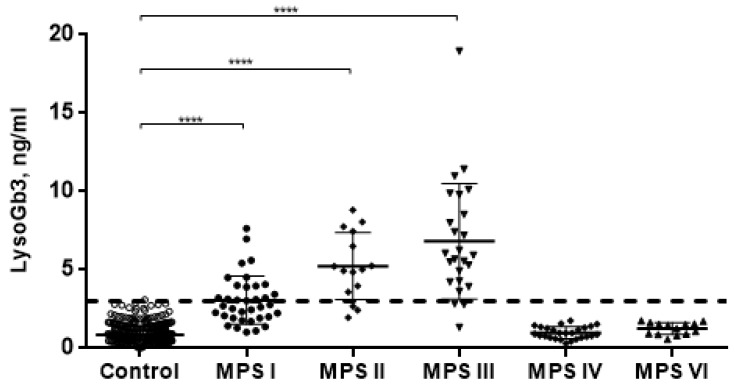
LysoGb3 concentration in normal controls and patients with different types of mucopolysaccharidoses (MPSs) (dotted line-cut-off for LysoGb3 level). **** *p* < 0.0001.

LysoGb3 concentration in 500 normal controls subjects was 0.86 ± 0.54 ng/mL. Untreated patients with MPS I, MPS II, and MPS III showed significantly elevated (*p* < 0.0001) LysoGb3 concentration (3.07 ± 1.55; 5.24 ± 2.13 and 6.82 ± 3.69 ng/mL, respectively). However, MPS I has a large number of intersections with the control group. Patients with MPS VI and MPS IVA, which are related to non-neuropathic forms, showed no elevation of LysoGb3 concentration (1.26 ± 0.39 and 0.99 ± 0.38 ng/mL, respectively) (Figure 1).

**Figure 2:**
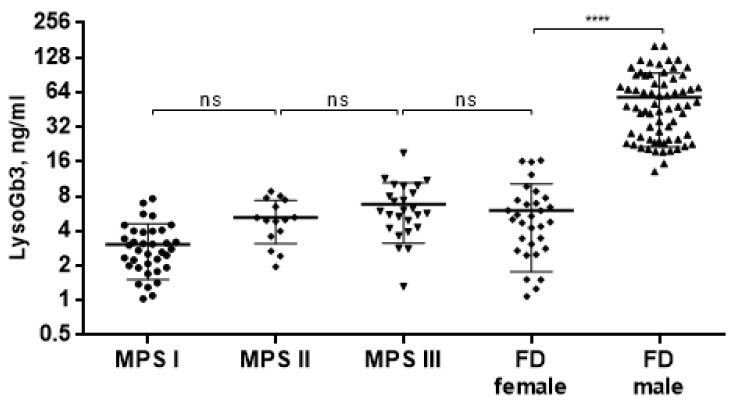
LysoGb3 concentration in patients with different types of MPSs and Fabry disease (FD) patients. **** *p* < 0.0001.

Therefore, we compared LysoGb3 concentration in untreated MPS patients and Fabry disease (FD) patients (male *n* = 68 and female *n* = 31) for whom this metabolite is a diagnostic marker (Figure 2). The LysoGb3 concentration in patients with untreated MPSs is comparable to the concentration of LysoGb3 in FD females (6.91 ± 6.07 ng/mL) but significantly lower compared to FD males (57.70 ± 36.18 ng/mL).

**Figure 3:**
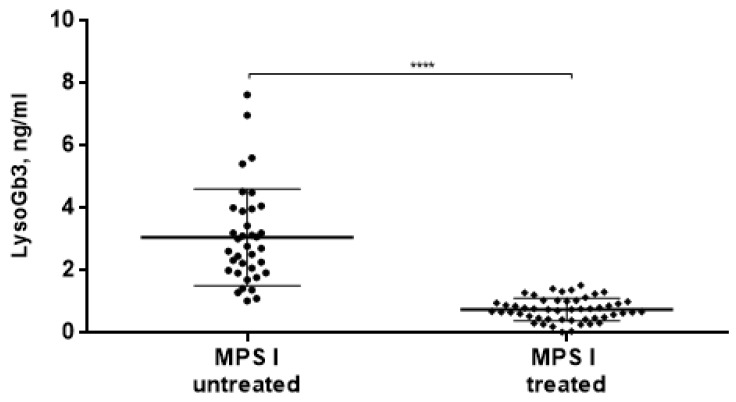
LysoGb3 concentration in MPS I patients before and on enzyme replacement therapy (ERT). **** *p* < 0.0001.

Moreover, we found that in MPS I patients who had been receiving ERT, the concentration of LysoGb3 significantly decreases (0.75 ± 0.36 ng/mL) compared to MPS I patients without ERT (3.07 ± 1.55 ng/mL) (Figure 3).

**Figure 4:**
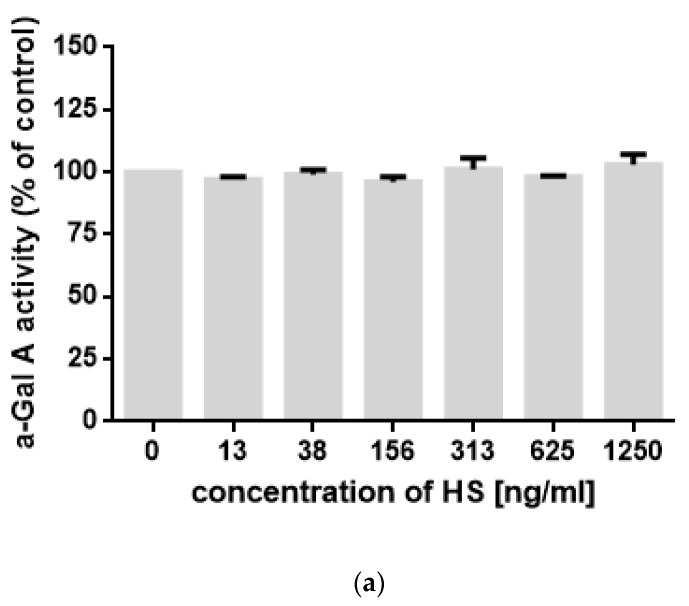

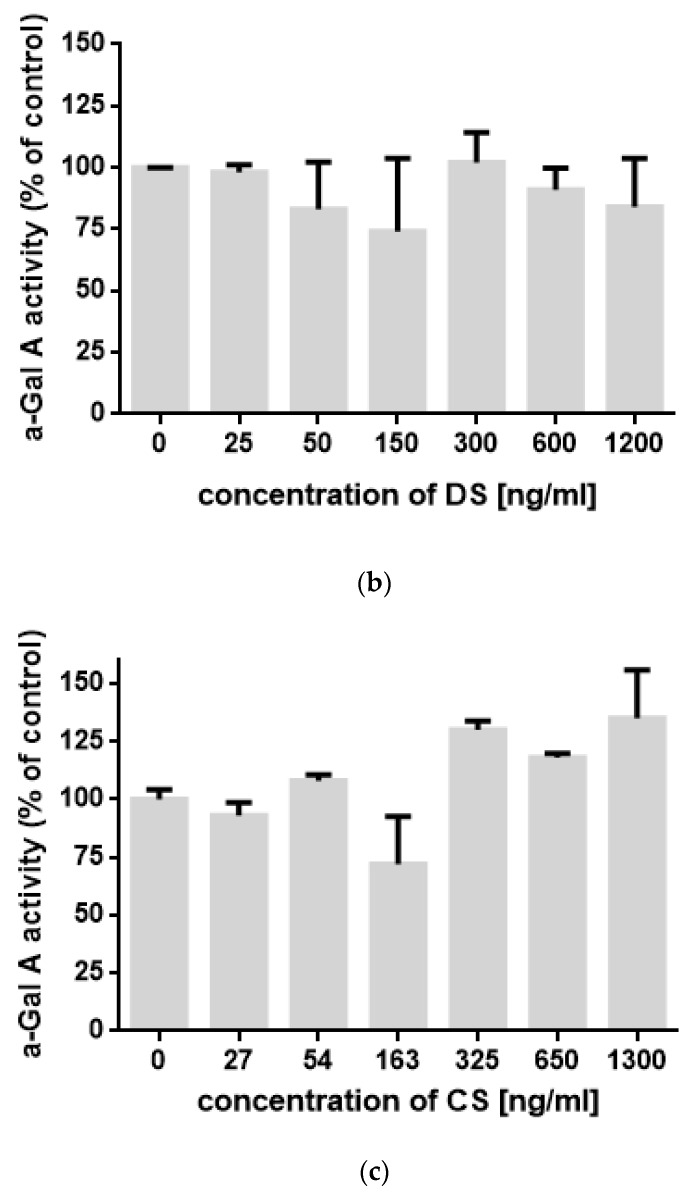
The activity of α-Gal A in the presence of different concentrations of (**a**) heparan sulfate (HS), (**b**) dermatan sulfate (DS), and (**c**) CS. Presented results are mean values from three independent experiments, with SD indicated as error bars. No statistically significant differences (at *p* < 0.05) were detected between control experiments (no GAGs added) and any measurements in the presence of GAGs.

To test if increased levels of LysoGb3 in MPS I, II, and III arise from direct impairment of the activity of alpha-galactosidase by HS, we have tested the activity of this enzyme in the presence of different concentrations of various GAGs. However, we found that HS, DS, and CS did not inhibit the enzyme activity in a wide range of concentrations (Figure 4). Therefore, we conclude that elevated levels of LysoGb3 arise as an indirect effect of HS storage rather than from direct inhibition of alpha-galactosidase activity by HS in lysosomes.

## 3. Discussion

The accumulation of additional compounds besides GAGs in MPSs, such as gangliosides and lactosylceramide (LacCer), especially in neuronopathic forms, is well known. However, the mechanism leading to their accumulation has not yet been found.

Some hypotheses have been proposed, but evidence to support these hypotheses remains to be established. The first hypothesis is based on the observation that accumulating GAGs can directly inhibit the activity of a variety of lysosomal enzymes [13,14] or cause some other critical disruption of the internal environment of late endosomes and lysosomes (pH change, lack of saposin-substrate interaction, etc.) leading to reduced degradation of additional substrates [15]. In our work, we have measured the activity of α-galactosidase A (α-Gal A) in vitro in the presence of different concentrations of HS and have not found changes in enzyme activity (Figure 4). Nevertheless, this does not exclude the effect of GAGs on the enzyme activity directly in the lysosomes. Additionally, we cannot exclude that HS may influence the expression of α-Gal A gene and other lysosomal enzymes genes. For further study, a series of experiments on cell cultures are planned.

**Figure 5:**
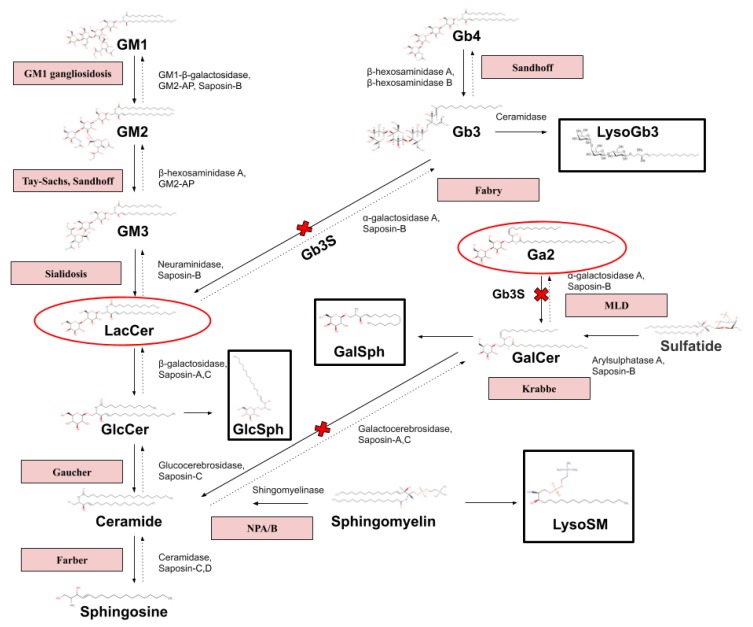
Pathway of sphingolipid degradation and related sphingolipidoses. (Miller et al [20] 2020; Heywood et al. [21] 2019).

Another theory of LysoGb3 accumulation in neuronopathic forms of MPSs may be the relationship between lactosylceramide (LacCer) and globotriaosylceramide (Gb3) (Figure 5). There is evidence that LacCer accumulates in MPS II and III [16,17], and it is the main core of most gangliosides [18]. It is also reported that LacCer is one of the main accumulated components in the brain samples of patients with neurological forms of MPSs; however, a significant increase of Gb3 was not fixed [19]. Globotriaosylceramide synthase (Gb3S) catalyzes the addition of α-1,4-galactose to lactosylceramide (LacCer), which produces globotriaosylceramide (Gb3), whose deacylated form is LysoGb3. In Fabry disease, accumulation of substrates containing terminal α-galactose: Gb3, LysoGb3, galabiosylceramide (Ga2/Gb2/Gal2Cer) has been reported [20,21]. In turn, galabiosylceramide (Ga2) is a structural isomer of LacCer.

The diseases are indicated in the red boxes. Lysosphingolipids (LysoSLs) are indicated in the black boxes. The red ovals contain structural isomers. Gb4—globotetraosylceramide; Gb3—globotriaosylceramide; LysoGb3—globotriaosylsphingosine; Ga2—galabiosylceramide; GalCer—galactosylceramide; GalCer—galactosylceramide; GalSph—galactosylsphingosine; LysoSM—lysosphingomyelin; GlcCer—glucosylceramide; LacCer—lactosylceramide; GM1, GM2, GM3—gangliosides; and GlcSph—glucosylsphingosine.

However, the pitfall of this theory is the substantial accumulation of LacCer in NPC, GM1, NPA/B, and in some other LSDs, but the level of LysoGb3 is not increased in these diseases. The accumulation of Lyso-LacCer, Lyso-GM1, Lyso-GM2, and Ga2 is also interesting for further studies.

Interestingly, the malfunction of the endocytic pathway may be demonstrated by adding a short analog of LacCer, which is transported to the Golgi under normal conditions. In fibroblasts obtained from some LSD patients (i.e., GM1 and GM2 gangliosidosis, prosaposin deficiency, metachromatic leukodystrophy, MPS IV, Fabry disease, Niemann-Pick disease, and Krabbe disease), it is misrouted and accumulates in late endosomes and lysosomes [22].

We have also shown that in MPS I patients, who had been receiving ERT, the concentration of LysoGb3 significantly decreases compared to MPS I patients without ERT. It appears that this metabolite can be used to track the effectiveness of ERT. Perhaps this dependence will also be observed in MPS II; unfortunately, we do not have data to confirm this hypothesis. However, the potential role of LysoGb3 for monitoring the treatment of MPSs, given the possibility of its determination in the blood, is quite promising. In assessment of efficacy of therapies for MPSs, it is useful to measure several different parameters, which should provide a more comprehensive view on the status of the patient, as any single indicator may be variable. As for LysoGb3 as a biomarker for diagnosis MPS I, proceeding on a large number of intersections with the control group, it is likely to bring no clinical benefit. Although 4 patients with the Hurler–Scheie phenotype have rather low LysoGb3 levels, it is impossible to use the metabolite to assess the severity of the disease in a group of 36 patients. This requires a larger number of patients with Hurler–Scheie and Scheie in the sample group. Additionally, further studies involving additional samples from naive patients would help to establish whether a correlation exists between the residual enzyme levels/severity of neurological symptoms and the LysoGb3 levels. Finally, in this study, the tendency of mild increase of LysoGb3 concentration was found in neuronopathic forms of MPSs. The task for the future is to examine the significance and reason for this observation.

## 4. Materials and Methods

### 4.1. Study Participants

The study included a total of 116 untreated patients with different types of MPSs: 36—MPS I (2.75 ± 2.13 y.a.), 15—MPS II (4.87 ± 2.50 y.a.), 15—MPS IIIA (7.73 ± 6.18 y.a.), 7—MPS IIIB (10.71 ± 9.59 y.a.), 3—MPS IIIC (11.33 ± 5.69 y.a.), 26—MPS IV (15,65 ± 14,89 y.a.), 14—MPS VI (20,07 ± 14,84 y.a.), and 51 treated MPS I patients with ERT. The control group comprised 250 healthy females (3.08 ± 2.74 y.a.) and 250 healthy males (3.43 + 2.55 y.a.). The group of patients with MPS I includes 4 patients with the Hurler-Scheie phenotype, 24—Hurler, and 8—no data. Criteria for a definite diagnosis of MPSs included a decreased or absent enzyme activity and mutation in the corresponding genes. All patients have provided informed consent for participation in the study that was approved by the ethics committee of Research Centre for Medical Genetics, Russia (the approval number 2015-5/3) and The Children’s Memorial Health Institute, Poland.

The DBSs were collected using standard procedures by puncturing the fingertip with a lancet and collecting the whole blood. Blood was dropped onto a 903 sample collection card (PerkinElmer^®^; Waltham, MA, USA), which was air-dried for approximately 2 h. The samples collected as EDTA whole blood were spotted onto the filter paper in the laboratory in Moscow using 50 μL of whole blood.

### 4.2. Sample Preparation

One disk (3.2 mm) was cut from each DBSs sample with a DBS puncher (PerkinElmer^®^; Waltham, MA, USA) and placed into wells of a 96 well microplate, and 100 μL of extraction solution [80:15:5 MeOH:ACN:H_2_O containing 10 ng/mL of IS] was added. The microplate was incubated for 1 h at 30 °C with orbital shaking (650 rpm). After incubation, 80 μL extracted mixture was transferred to a new microplate. Then, 10 μL was injected in the LC–MS/MS system [23].

### 4.3. Measurement of LysoGb3 Concentration

The lysosphingolipid measurements (LysoGb3 [Fabry Disease], LysoSM [Niemann–Pick disease types A/B], LysoSM-509 [Niemann–Pick disease types A/B and C], and hexosylsphingosine [Gaucher and Krabbe Disease]) were performed by reverse-phase liquid chromatography using the HPLC Shimadzu Nexera (Shimadzu, Kyoto, Japan) and the XBridge™ BEH C18 column 2.1 × 50 mm with a 3.5 μm particle size (Waters, Milford, MA, USA). Mass spectrometry detection was conducted with an ABI SCIEX 5500 QTrap (AB SCIEX, Concord, ON, Canada) set in the positive mode using an electrospray ionization source (ESI). LC–MS/MS analysis was performed as previously described [23].

### 4.4. Measurement of α-Gal A Activity In Vitro

The activity of α-Gal A has been determined as described previously [24], using a purified form of the enzyme (Fabrazyme, Sanofi-Genzyme). Purified HD, DS, and CS were purchased from Merck.

### 4.5. Statistical Analysis

Statistical analysis was performed using the GraphPad Prism software (GraphPad Software Inc., San Diego, CA, USA). Data are expressed as medians and standard deviations. The nonparametric two-tailed Mann–Whitney U test was used to evaluate the differences between groups. Statistical significance was estimated using multiple comparisons. A *p*-value < 0.05 was considered statistically significant.

## 5. Conclusions

In this study, we reported that LysoGb3 could be a secondary biomarker for the diagnosis of MPSs types associated with the accumulation of HS and used to track therapy effectiveness. Moreover, we showed that DBS could be used for selective screening for the diagnosis of severe neuronopathic forms of MPS and, potentially, also for therapeutic monitoring. However, further studies are needed to verify our findings and to determine the role of LysoGb3 in MPSs pathogenesis.

## Abbreviations

MPSs	Mucopolysaccharidoses
GAGs	Glycosaminoglycans
CNS	Central nervous system
HS	Heparan sulfate
LacCer	Lactosylceramide
Gb3	Globotriaosylceramide
LysoGb3	Globotriaosylsphingosine
DBSs	Dried blood spots
HPLC-MS/MS	High pressure liquid chromatography–tandem mass spectrometry
LSDs	Lysosomal storage disorders
LysoSLs	Lysosphingolipids
α-Gal A	α-galactosidase A
